# Error assessment of subjective estimates of linear breast dimensions versus the objective method

**DOI:** 10.3205/000333

**Published:** 2024-07-08

**Authors:** Parthena Karavasili, Helga Henseler

**Affiliations:** 1 Klinik am Rhein, Klinik für Plastische und Ästhetische Chirurgie, Düsseldorf, Germany

**Keywords:** subjective estimates, linear breast dimensions, objective method, reproducibility, accuracy, errors, simplified breast shape analysis, Vectra Camera System

## Abstract

**Objective:**

The study aimed to investigate the subjective method of estimating linear breast dimensions in comparison to the objective method.

**Methods:**

The reproducibility and accuracy of the subjective method of estimating linear breast dimensions during a simplified breast shape analysis were examined. Four linear breast dimensions including the distance from the sternal notch to the nipple, distance from the nipple to the inframammary fold, distance from the nipple to the midline and under-breast width were evaluated based on subjective estimates. Images from 100 women with natural breasts and without any history of breast surgery were reviewed by two examiners three times each. The cases were obtained from a large database of breast images captured using the Vectra Camera System (Canfield Scientific Inc., USA). The subjective data were then compared with the objective linear data from the Vectra Camera System in the automated analysis. Statistical evaluation was conducted between the three repeated estimates of each examiner, between the two examiners and between the objective and subjective data.

**Results:**

The intra-individual variations of the three subjective estimates were significantly greater in one examiner than in the other. This trend was consistent across all eight parameters in the majority of the comparisons of the standard deviations and variation coefficients, and the differences were significant in 14 out of 16 comparisons (p<0.05). Conversely, in the comparison between the subjective and objective data, the estimates were closer to the measurements in one examiner than the other. In contrast to the reproducibility observed, the assessment of the accuracy revealed that the examiner who previously presented with less reproducibility of the estimated data overall showed better accuracy in comparison to the objective data. The overall differences were inconsistent, with some being positive and others being negative. Regarding the distances from the sternal notch to the nipple and breast width, both examiners underestimated the values. However, the deviations were at different levels, particularly when considering the objective data from the Vectra Camera System as the gold standard data for comparison. Regarding the distance from the nipple to the inframammary fold, one examiner underestimated the distance, while the other overestimated it. An opposite trend was noted for the distance from the nipple to the midline. There were no differences in the estimates between the right and left sides of the breasts. The correlations between the measured and estimated distances were positive: as the objective distances increased, the subjective distances also increased. In all cases, the correlations were significant. However, the correlation for the breast width was notably weaker than that for the other distances.

**Conclusions:**

The error assessment of the subjective method reveals that it varies significantly and unsystematically between examiners. This is true when assessing the reproducibility as well as the accuracy of the method in comparison to the objective data obtained with an automated system.

## Zusammenfassung

**Hintergrund:**

Die Studie zielte darauf ab, die subjektive Methode des Schätzens linearer Brustdimensionen im Vergleich zur objektiven Methode zu untersuchen.

**Material und Methode:**

Die Wiederholbarkeit und Genauigkeit der subjektiven Methode, lineare Brustdimensionen bei einer vereinfachten Formanalyse zu schätzen, wurde untersucht. Vier lineare Brustdimensionen unter Einschluss der Distanz vom Jugulum zum Nippel, Distanz vom Nippel zur Unterbrustfalte, Distanz vom Nippel zur Mittellinie und die Unterbrustweiten wurden durch subjektive Einschätzungen beurteilt. Zu diesem Zweck wurden Bilder von 100 Frauen mit natürlichen Brüsten und ohne irgendwelche Brustoperationen durch zwei Untersucherinnen jeweils drei Mal betrachtet. Die Fälle wurden aus einer großen Datenbank von Brustbildern entnommen, die mittels des Vectra Camera Systems (Canfield Scientific Inc. U.S.A) aufgenommen wurden. Der Vergleich der subjektiven Daten wurde zu den objektiven linearen Daten des Vectra Camera Systems gemäß der automatisierten Analyse gezogen. Die statistische Auswertung wurde zwischen den drei Wiederholungsschätzungen jeder Untersucherin, zwischen den beiden Untersucherinnen und zwischen den objektiven und subjektiven Daten durchgeführt.

**Ergebnisse:**

Die intraindividuellen Variationen der drei subjektiven Schätzungen waren signifikant höher bei einer der beiden Untersucherinnen als bei der anderen. Dieser Trend war durchgehend vorhanden bei allen acht Parametern in der Mehrheit der Vergleiche der Standardabweichungen und Variationskoeffizienten, und die Unterschiede waren signifikant bei 14 von 16 Vergleichen (p<0,05). Im Gegensatz dazu ergab sich beim Vergleich der subjektiven mit den objektiven Daten, dass die Schätzungen bei einer der Untersucherinnen näher an den Messwerten waren als bei der anderen. Im Gegensatz zur beobachteten Reproduzierbarkeit ergab die Untersuchung der Genauigkeit, dass die Untersucherin, die sich vormalig mit geringerer Reproduzierbarkeit der geschätzten Daten zeigte, insgesamt eine bessere Genauigkeit beim Vergleich zu den objektiven Daten aufwies. Die Unterschiede ergaben sich insgesamt als unsystematisch, teils positiv, teils negativ. Für die Distanzen Jugulum zum Nippel sowie Brustbasisbreite unterschätzten beide Untersucherinnen die Werte. Allerdings waren die Abweichungen auf unterschiedlichem Niveau, insbesondere unter der Annahme, dass die objektiven Messdaten des Vectra Systems den Goldstandard zum Vergleich darstellten. Für die Distanz Nippel zur Unterbrustfalte schätzte die eine Untersucherin die Distanz zu niedrig und die andere zu hoch. Das Gegenteil traf für die Distanz Nippel zur Mittellinie zu. Es gab keine Unterschiede bei den Schätzungen in Bezug auf die rechte oder linke Seite der Brust.

Die Korrelationen zwischen den gemessenen und geschätzten Strecken waren positiv: Mit steigenden objektiven Werten wurden die Schätzungen ebenfalls größer. In allen Fällen waren die Korrelationen signifikant. Allerdings war die Korrelation für die Brustbasisweite deutlich schwächer als jene für die anderen Strecken.

**Schlussfolgerung:**

Die Fehleranalyse der subjektiven Methode ergibt, dass diese signifikant und unsystematisch zwischen Untersuchern variiert. Dies ist wahr bei der Untersuchung sowohl der Reproduzierbarkeit als auch der Genauigkeit der Methode im Vergleich zu den objektiven Daten, die mit einem automatisierten System ermittelt wurden.

## Introduction

To date, it is still common in daily clinical practice to apply subjective judgements of volumes, shapes and surfaces when discussing breast-related issues among female patients [1], [2], [3], [4], [5]. However, judgements and opinions can differ among individuals over time [6]. Consequently, questions regarding the validity of subjective assessments arise. As an advancement from a purely subjective method of breast assessment, the utilization of manual tape measurements for assessing linear distances and adopting a two-dimensional (2D) approach has gained acceptance in breast clinics worldwide [7], [8], [9], [10]. However, in daily clinical practice, manual tape measurements are predominantly used preoperatively, while subjective estimates are employed intraoperatively and postoperatively.

In the field of breast surgery, different aspects, such as breast volumes, shapes and surfaces, have been investigated [11]. Various methods have been applied for breast assessment [12]. A previous study focused on analyzing the validity of subjective estimates of breast volume [13] in comparison to objective measurements. For this purpose, the objective breast analysis tool developed by Glasgow University was utilized. It was found that the subjective method resulted in a significant underestimation of breast volumes with considerable variations between individual cases. Examiners overestimated smaller breast volumes and underestimated larger breast volumes. Further, the reproducibility of the subjective method was less than that of the objective method. The sizes of the errors increased with increasing breast sizes. While the assessment of breast volume involves an analysis of three-dimensional (3D) values, the analysis of breast shape appears to be even more complex. The latter involves multiple variable geometric data of the breast surface and should be differentiated from the assessment of breast volume [11]. To provide a more applicable approach for clinical practice, another prior study presented a simplified method that relied on four key linear breast dimensions [14]. While breast shape analysis appears to be more challenging than breast volume assessment, the author noted that it was possible to conduct a meaningful and simplified assessment using a 2D approach involving four linear distances. Three of these linear distances relied on the position of the nipple and the other distance on the breast width. The analysis revealed that the linear distance from the nipple to the midline seemed important, as it showed the largest deviations in natural breasts between both sides. However, this linear distance is frequently neglected in daily clinical practice, where it is common to assess only vertical measurements, such as the distances from the sternal notch (also called the jugulum) to the nipple and from the nipple to the inframammary fold. Therefore, the term ‘aesthetic triangle of the breast’ was introduced, built from the distances between the jugulum and the midline to the nipples [14]. In the simplified breast shape analysis in the prior study, this aesthetic triangle was found to be especially relevant. Following this investigation, the question regarding the validity of the subjective method of estimating 2D distances in simplified breast shape analyses arose. An error assessment of the subjective method of estimation currently proves to be of utmost interest. A study was then conducted in this field [15], where the inter-observer variability of judging the aesthetic outcome of patients with breast cancer was described. In comparison to this previous study, the present study focuses on breast shape analyses of natural breasts without previous surgery as the subject of interest.

## Objective

This study aimed to investigate the subjective method of estimating linear breast dimensions in comparison to the objective method.

## Methods

**Figure 1:**
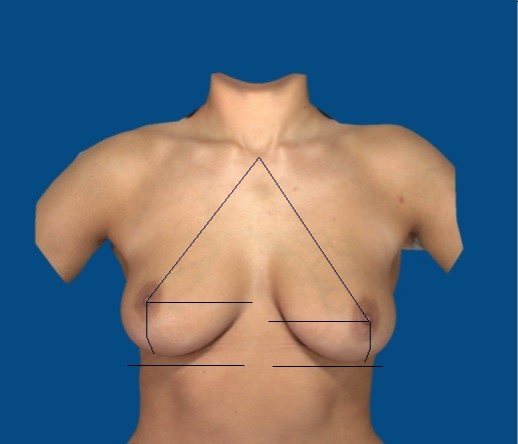
Four linear distances in female breasts

**Figure 2:**
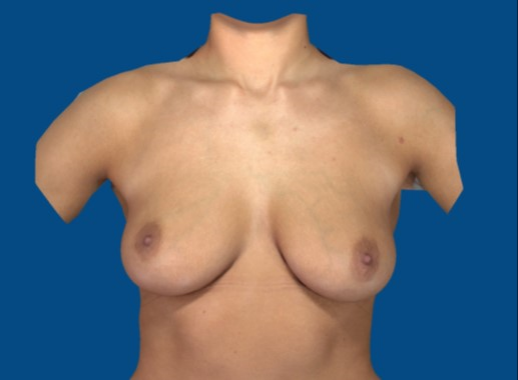
Digital image of natural breasts without markings or any apparent application of automated software analysis obtained using the 3D Vectra Camera System for subjective estimations of linear distances

The reproducibility and accuracy of the subjective method of estimating linear breast dimensions during a simplified breast shape analysis were examined. Four linear breast dimensions including the distance from the sternal notch to the nipple, distance from the nipple to the inframammary fold, distance from the nipple to the midline and under-breast width were evaluated based on subjective estimates (Figure 1 (Fig. 1)). For this purpose, images from 100 women with natural breasts, devoid of any history of breast surgery, markings or any apparent application of objective software analysis, were reviewed by two examiners three times each (Figure 2 (Fig. 2)). The two examiners subjectively estimated the linear distances. The one examiner was a consultant general surgeon with training in plastic surgery, the other examiner was a consultant plastic and aesthetic surgeon. 

The cases were retrieved in alphabetical order from a large database of breast images collected using the 3D Vectra Camera System (Canfield Scientific Inc., USA) at a plastic surgery clinic. 

For the assessment of the reproducibility of the subjective method the intra-individual variations were calculated. The intra-individual variations were based on the standard deviation of the three estimations of each examiner. In all 100 images, the mean, standard deviation, median, minimum and maximum values as well as the variation coefficients of the distances were calculated. Further, a comparison between both examiners was drawn. 

For assessment of the accuracy of the subjective method a comparison between subjective and objective data was conducted. The subjective data of the estimations were compared with the objective linear data provided by the Vectra Camera System in the automated analysis. In general, the Vectra Camera System utilizes automated landmark positioning on digital images obtained after capture via software processes (https://www.canfieldsci.com/). The objective data from the Vectra Camera System were taken as the gold standard data for comparison. Statistical evaluation was conducted between the three repeated estimations of each examiner, between the two examiners and between the subjective and objective data. Differences were calculated.

The standard deviations were based on the distribution of the individual estimations around the means. The standard deviations were compared between the examiners. The variation coefficients were calculated to evaluate the intra-individual variations independently of the obtained values. Generally, variation coefficients reflect the relative variability of a parameter and are presented as percentages. They describe how much data spread around means.

The variation coefficients were computed by dividing the standard deviation by the mean, yielding a percentage value in each case relative to the mean. 

The differences were calculated between the objective data and the mean of the three subjective estimates in centimetres. Negative values indicated that the estimates were larger than the measurements. The differences were calculated for both examiners.

For the statistical analysis, the following software programmes were used: BiAS for Windows [16] and several packages of R [17], including the ggplot2 package for creating images. 

For the investigation of the relationship between the estimated and measured distances, Spearman’s correlation coefficients (rho) were utilized. Spearman’s rho is a measure of the quality of correlations and is suitable for assessing dependent, arbitrarily distributed, continuous variables.

Rho can range from –1 to +1. A larger value indicates a stronger correlation. In this study, squared correlation coefficients (rho^2^) were also assessed to evaluate statistical certainty. Rho^2^ describes the proportion of the variance in the target figure that is explained by the independent variable. A rho^2^ of about 10% (equalling a value of ±0.32) indicates a weak correlation; 50% (±0.71), clear correlation; and 1.0 (>0.71), linear correlation.

The dependent variables were evaluated among the same cases: the measured and estimated linear distances were assessed in the same digital images. Thereafter, statistical significance was determined [18].

The Wilcoxon test for paired differences was used to assess the statistical significance of differences. This approach was deemed preferable to the t-test owing to the absence of assumptions regarding the distribution function of both variables. 

## Results

### Reproducibility: intra- and inter-individual variations of the subjective estimates of distances

Table 1 (Tab. 1) displays the intra-individual standard deviations and variation coefficients for both examiners as well as the inter-individual differences based on the p-values calculated using the Wilcoxon matched-pairs test. 

**Figure 3:**
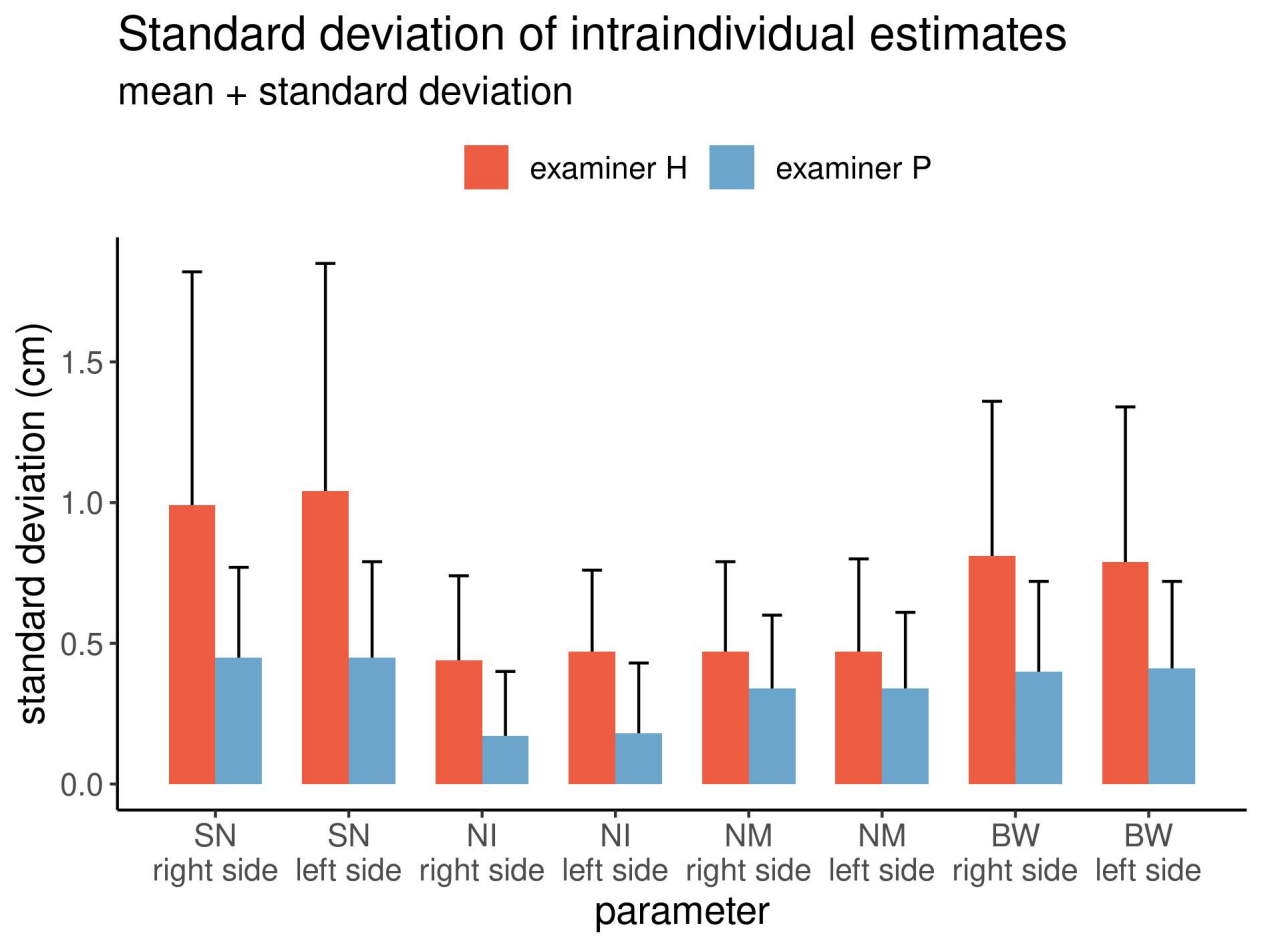
Intra-individual variations of the estimated distances by both examiners

The intra-individual variations of the estimates for both examiners are graphically displayed in Figure 3 (Fig. 3). 

The standard deviations of all estimated linear distances differed, with one examiner performing better in subjectively estimating the distances than the other. This trend was consistent across all eight linear distances estimated. A similar pattern was also noted among the variation coefficients (Table 1 (Tab. 1)). The differences were significant in 14 out of the 16 comparisons (p<0.05). 

### Accuracy: differences between the estimated and measured distances and between the examiners

The mean differences between the three distances estimated by both examiners and the objectively measured distances via software analysis are displayed in Table 2 (Tab. 2). 

**Figure 4:**
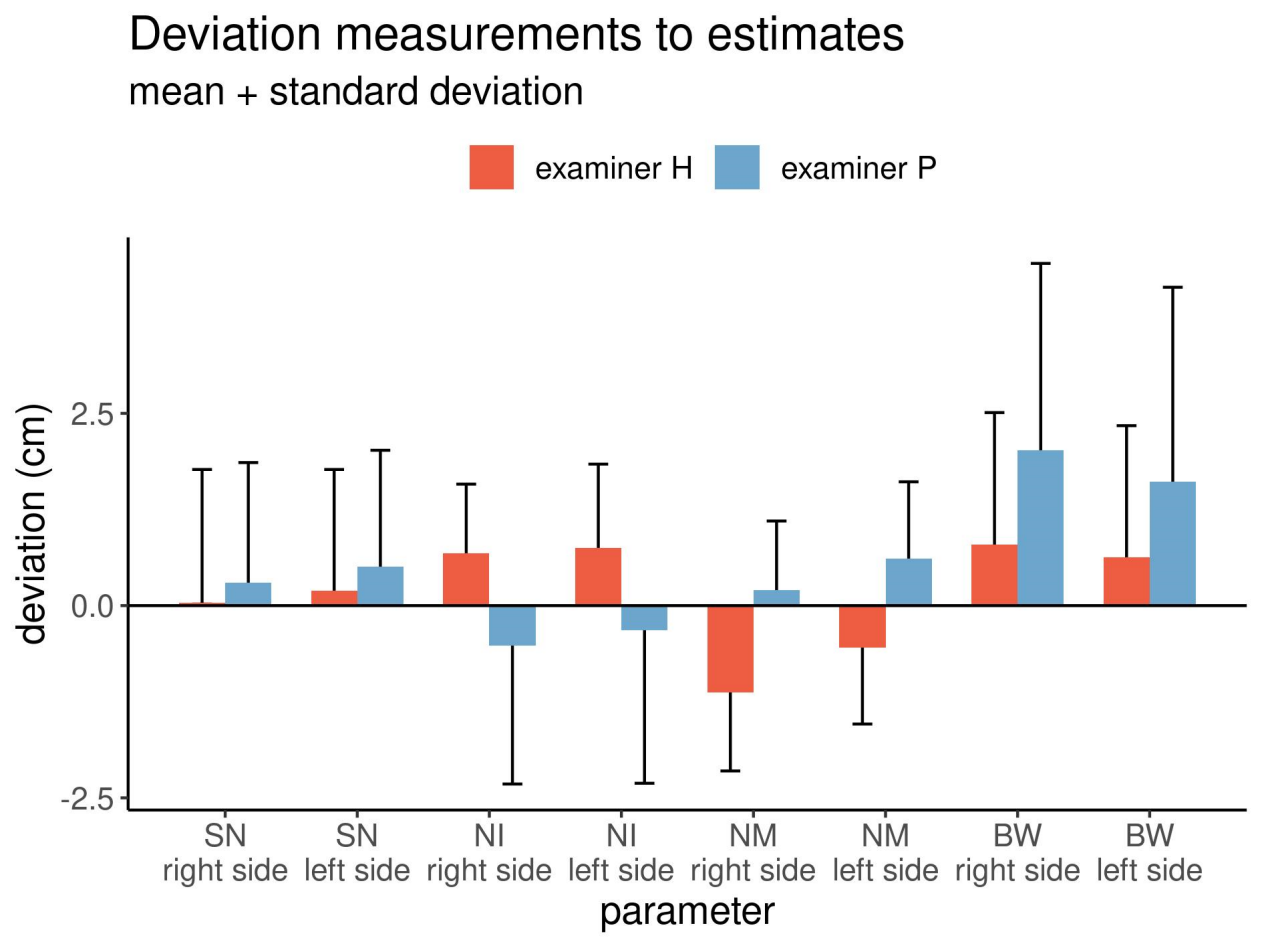
Mean differences between the measured and estimated values and between the examiners

The differences between measurements and estimates for both examiners are graphically displayed in Figure 4 (Fig. 4). 

In all comparisons, the differences were significant (Wilcoxon matched-pairs test: p<0.05). The differences between the left and right sides of the breasts were comparable. The mean differences in the distance from the sternal notch to the nipple between the left and right sides were positive for both examiners. On average, both examiners underestimated this distance, but with examiner P estimating a slightly but significantly shorter distance than examiner H. Regarding the distance from the nipple to the inframammary fold, examiner H underestimated the distance by 0.7 cm, while examiner P overestimated it by 0.3–0.5 cm. Regarding the distance from the nipple to the midline, examiner H overestimated the distance, while examiner P underestimated it. Finally, regarding the breast width, both examiners underestimated the width, but the estimate by examiner P was shorter than that by examiner H.

Overall, the standard deviations of the estimated and measured distances were inconsistent, with some being positive and others being negative. However, each examiner showed the same tendency of deviations for opposing distances on the right and left sides of the breasts.

### Correlation: relationship between the measured and estimated distances for both examiners

The correlation coefficients between the eight measured and estimated distances for both examiners as well as the levels of significance are shown in Table 3 (Tab. 3).

**Figure 5:**
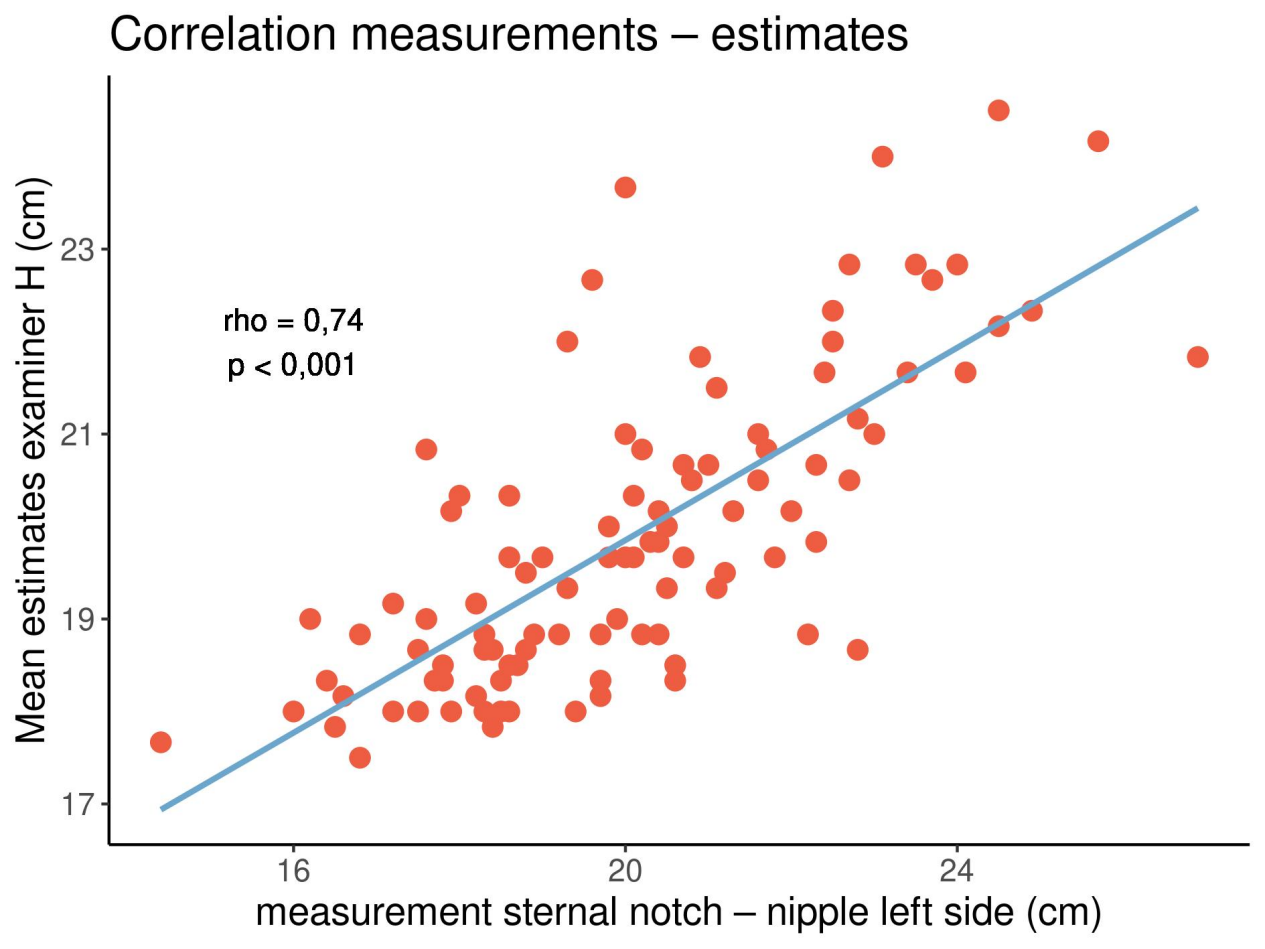
Correlation between the measured and estimated distances from the sternal notch to the nipple on the left side of the breasts for examiner H

**Figure 6:**
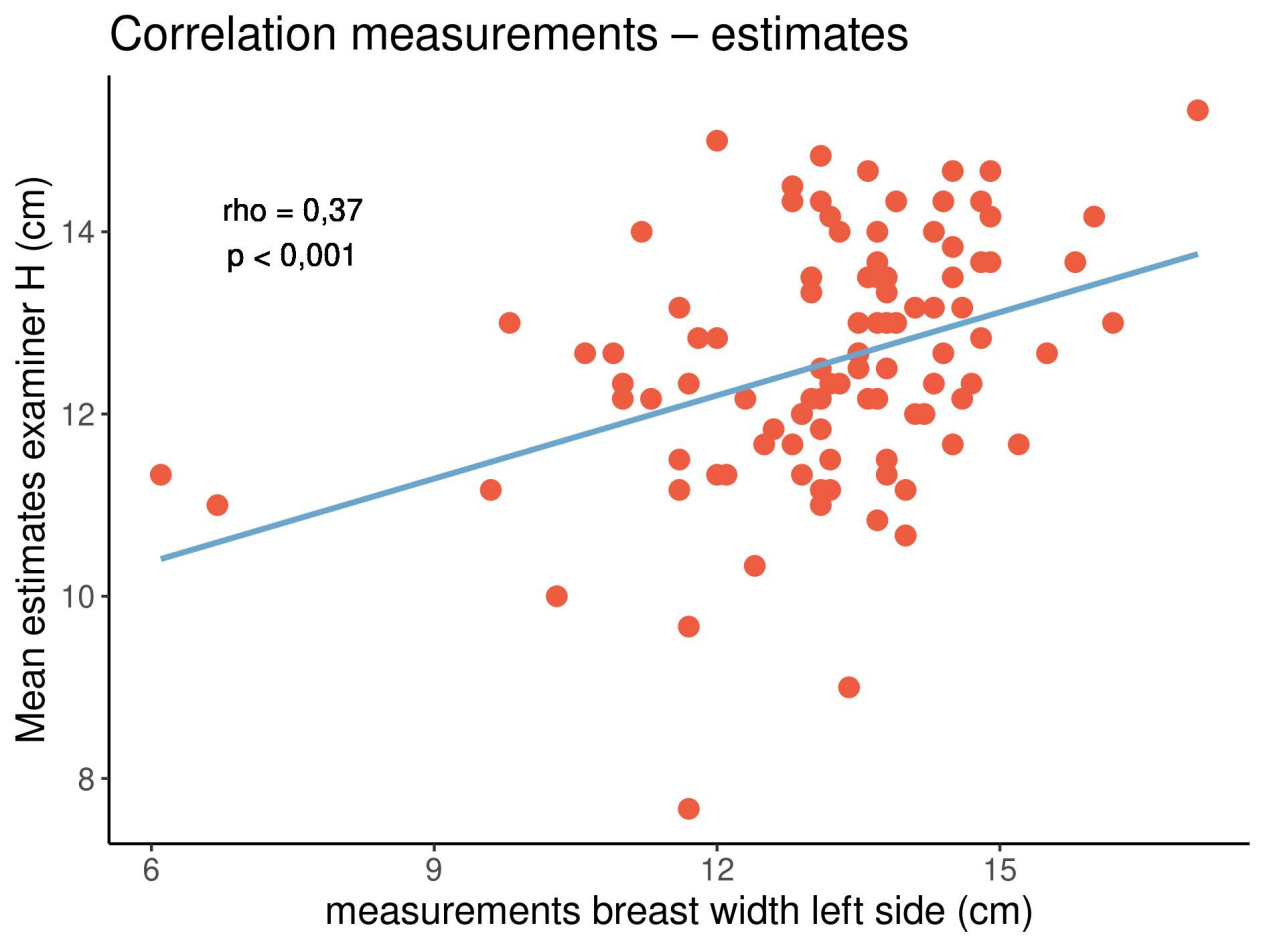
Correlation between the measured and estimated breast widths on the left side of the breasts for examiner H

**Figure 7:**
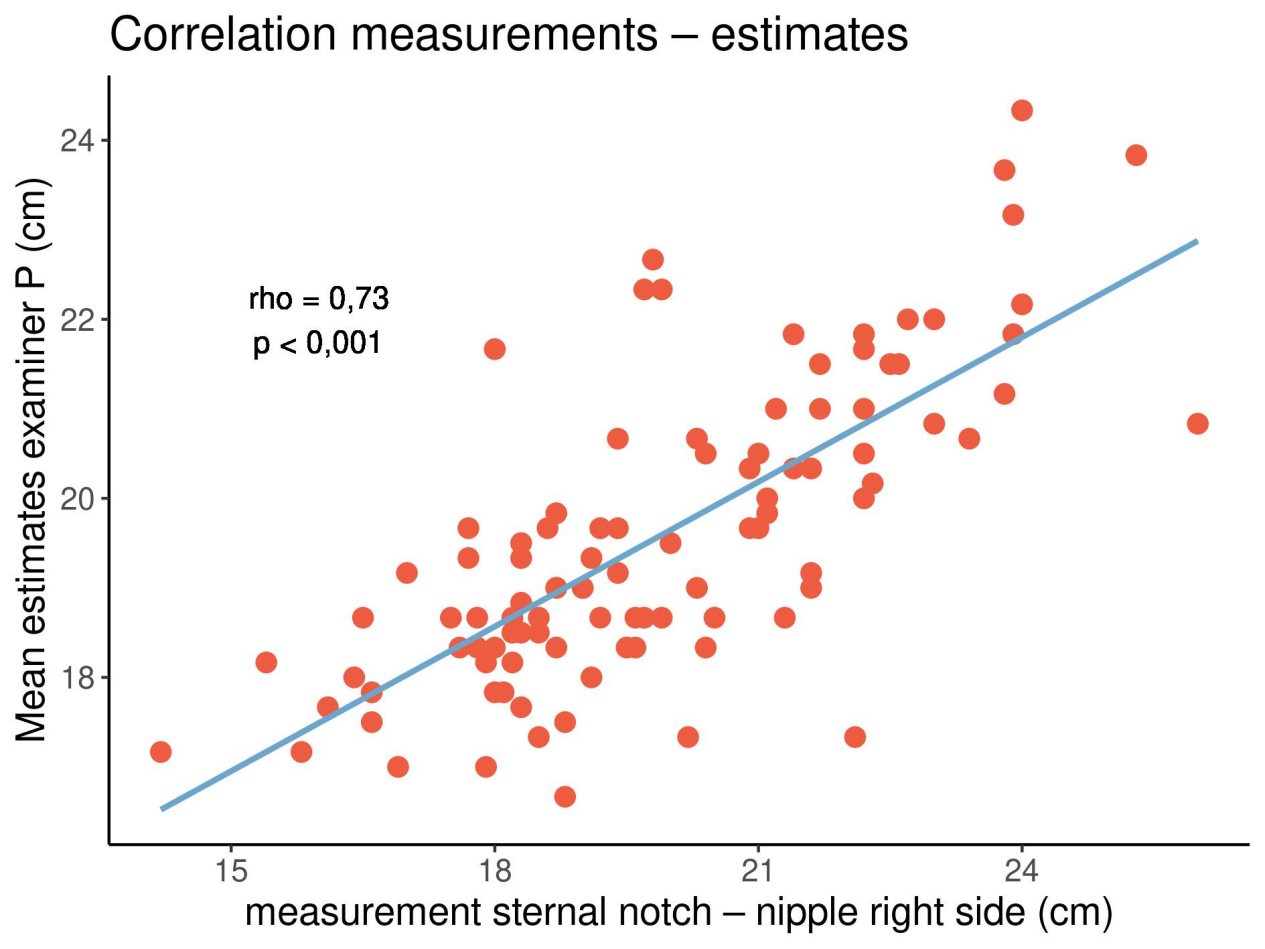
Correlation between the measured and estimated distances from the sternal notch to the nipple on the right side of the breasts for examiner P

All correlation coefficients were positive, with values ranging from 0.27 to 0.74, indicating that both parameters tended to show the same orientation. In particular, longer distances tended to be estimated longer, while shorter distances tended to be estimated shorter. All correlations were significant (p<0.05). The highest and lowest correlation coefficients were noted in the distance from the sternal notch to the nipple (rho=0.66–0.74) and the breast width (rho=0.27–0.43), respectively. There were no clear or consistent differences between both examiners and between the left and right sides of the breasts. Figure 5 (Fig. 5), Figure 6 (Fig. 6) and Figure 7 (Fig. 7) display three out of the 16 correlations.

In both examiners and on either side of the breasts, there was a strong correlation between the estimated and measured distances from the sternal notch to the nipple. On the contrary, there was a weaker correlation between the estimated and measured breast widths.

## Discussion

The present study evaluated the subjective method of estimating four linear breast dimensions, three of which relied on the position of the nipple and the other on the breast width. These four linear distances were presented in a previous work [14]. In the assessment of the intra-observer and inter-observer errors, one examiner showed significantly less variability than the other. The subjective method therefore revealed different results in different examiners in the assessment of the reproducibility of the method. Further, in the present study, there were differences noted between the measured and estimated linear breast dimensions among the same cases at different time points and between the two examiners. In contrast to the reproducibility observed, the assessment of the accuracy revealed that the examiner who previously presented with less reproducibility of the estimated data overall showed better accuracy in comparison to the objective data. The subjective data, along with their accuracy, were found to vary, with both underestimations and overestimations of the linear distances noted between the examiners. The objective data were obtained via modern 3D imaging using the Vectra Camera System. Finally, the correlation analysis revealed that all subjective and objective data somewhat correlated with each other. This finding is in contrast to a previous report that the breast volumes in small and large breasts are subjectively overestimated and underestimated, respectively [13]. The reason for this difference remains unknown, but it could be postulated that breast volumes as 3D figures might be more difficult to grasp than linear breast dimensions as 2D figures. In the current study, the overall differences varied, with limited reproducibility and accuracy with underestimations and overestimations of the data. These findings highlight the weakness of the subjective method.

While the present study focused on natural breasts, other studies have aimed to quantify the optimal nipple position in mastopexy and breast reduction procedures. The breast base width has also been utilized for the definition of a proportional nipple-to-inframammary fold distance for optimal breast aesthetics [19]. The present study also investigated the breast width beyond merely an analysis of the position of the nipple–areola complex, as the breast width was also considered a parameter of importance in the simplified breast shape analysis. While previous researchers have described the initial steps necessary for a skin envelope design, we presented a clear and simple approach of evaluating linear breast dimensions that can be easily understood, copied and used for breast assessments [14].

For a long time, there has been no consensus on the application of breast assessment methods. Therefore, attempts have been made to present a standardized anthropometric protocol for such assessment [20]. However, critics have expressed the need to validate anthropometric studies and highlighted the difficulties in conducting studies for this purpose, including its impracticality [1]. The reasons for such views lie in the need to conduct large-scale studies to validate anthropometric measures with several observers, who would need to measure the same subjects multiple times. The potential role of 3D imaging is outlined as a possible solution of this conflict. However, while quantitative measures from digital imaging seem to yield acceptable outcomes, there are possible limitations. Therefore, subjective, anthropometric and digital methods continue to be utilized.

In the present study, 3D imaging was conducted as the objective method of measuring the linear distances. Currently, one of the most advanced 3D imaging tools is the Vectra Camera System. In a recent study, the reliability and reproducibility of this system in comparison to those of the Harvard Cosmesis Scale were investigated [21]. The authors concluded that the Vectra Camera and Mirror Software have the potential to objectively assess breast symmetry, but their use was judged to be unideal. The subjective assessment using the Harvard Cosmesis Scale yielded no-to-moderate intra-observer agreement and weak inter-observer agreement, similar to the present findings on the subjective method.

Breast surgery has recently been described as one of the most important aesthetic and reconstructive treatments. However, a previous study outlined that no ideal method for outcome assessments would exist [22]. The subjective method of evaluation was seen as one method with the greatest difficulties. Therefore, four different objective computer/software systems were examined, one of which was developed by the authors. The positive aspects of symmetry assessment of this novel system were outlined based on an optical flow algorithm.

The same study group further expressed that in aesthetic breast surgery, the subjective assessment of the surgeon or the patient was considered the gold standard method of breast assessment [23]. In contrast, different objective measurement methods were described to be available for symmetry assessment in conservative breast surgery. The study was then considered to have newly explored the use of an objective symmetry assessment method in the field of aesthetic breast surgery. The authors concluded that the new objective method via software analysis has less variability than the subjective method. This conclusion is similar to the present findings although the methodology between the studies differs.

In line with the previously described importance of the subjective method of breast assessment as the gold standard approach in aesthetics [23], we aimed to investigate the validity and accuracy of this method in comparison to those of the objective method as well as the intra-observer and inter-observer errors. For comparison, we selected the Vectra Camera System to conduct the objective method. In general, this system aims to support surgeons in the field of aesthetic breast evaluation and simulation for breast augmentation cases. Several studies on the usage of the Vectra Camera System have recently been published [24], [25], [26], [27], [28]. Initially, the 3D imaging system has been seen as an emerging technology in many breast augmentation practices [24]. It has been demonstrated to be able to measure breast volumes, although the values are smaller than those obtained using magnetic resonance imaging technology [25]. However, measurements have revealed a linear association and been described to have excellent reliability. The calculated 3D breast volume does not significantly differ from the estimated volume.

Another study group investigated the influence of the number and position of markers on the skin for image synthesis on the accuracy of measurements [26]. Setting appropriate marker conditions was viewed as important for distance measurements to achieve clinically satisfactory accuracy. In the present study, the number and position of skin markers were set via automated software interaction, which led to excellent reproducibility in the automated analysis of the linear distances. After the initial capture of the images, the software analysis with automated landmark positioning and the following analysis revealed an exact repetition of the data after reloading of the digitized images at a later date. Owing to the automated landmark positioning of the objective system, the reproducibility of the data in the automated analysis after the initial capture was considered excellent. In contrast, this trend was not observed with the subjective method.

In a recent study, the Vectra Camera System was utilized for anthropometric breast measurements in a mannequin model [27]. The camera was judged to be a reliable and reproducible tool. However, the study group was small, and the mannequin model was deemed to differ from real-life conditions.

Bai et al. evaluated whether 3D imaging could be the gold standard method for breast symmetry and aesthetic assessments [28]. The authors highlighted the lack of an accurate standardized objective method for aesthetic outcome assessment after breast surgery. Two observers analyzed 3D images captured using the Vectra Camera System in 58 women after mastectomy and immediate reconstruction and obtained 348 measurements relative to breast symmetry and 696 measurements relative to breast volume. While the intra-observer reproducibility was found to range from substantial to excellent, the inter-observer reproducibility was less than the intra-observer reproducibility. The authors concluded that 3D surface imaging in its current form is not an excellent method of assessment for breast symmetry.

In a different study conducted among 40 patients, a handheld digital imaging device – the Artec Eva device – was used for assessment during breast surgery [29]. The authors found no significant difference between manual measurements obtained using a tape measure and digital measurements obtained using the imaging device. In another study, a prototype software was developed for automated digital anthropometry and compared with manual measurements of 46 breasts [30]. In six out of seven measurements, no significant differences were observed. While this trial validated the digital anthropometry method, there was a discrepancy in one out of the seven measurements, raising questions about the findings of this study.

Possible limitations in establishing the defining points of the breast fold as well as in determining the lower portion and lateral extension of the breasts from images of the frontal, left and right views have been previously described [31]. These limitations in the defining points for anthropometric measurements of the breast can influence the accuracy and reproducibility of measurements. Therefore, the Vectra Camera System applies an automatized digitization of these points whenever possible to avoid possible errors. This aspect supports the use of the Vectra Camera System as an objective method for comparison with the subjective method. Nonetheless, there remain limitations in ptotic breast shape analyses, in which automated landmark positioning encounters challenges.

A prior study determined what measurements reveal in aesthetic breast surgery [32]. Standard tape measurements were applied by a single surgeon for evaluating linear distances. The borders of the breast footprint expanded with the addition of an implant and reduced after excision of breast tissue.

Direct anthropometric measurements of the breast typically carry the need to trace body curves, which has been criticized as introducing bias into linear measurements. Therefore, a self-designed web application – BreastIdea – was presented in a previous study [33]. This application was seen as a reliable tool for indirect breast measurements. Similarly, the Vectra Camera System utilized in the present study provided software-driven anthropometric measurements for indirect breast assessments. In their study, Isaac et al. compared indirect measurements from 3D imaging and analysis with direct anthropometric measurements and evaluated the reliability of the measurements among four raters [34]. The authors found that static measurements such as linear distances towards the nipple were substantially reliable, while dynamic measurements such as the anterior pull skin stretch, distance from the nipple to the inframammary fold under maximal stretch or soft tissue pinch thickness at the upper or lower pole were not.

Taken together, the abovementioned studies reveal that any method, even if perceived as an advancement over another method, such as indirect anthropometry over direct anthropometry or direct anthropometry over subjective estimation, has limitations. Nevertheless, research into this topic continues. In a recent study, the reliability of the BCCT.core software in evaluating breast appearance was examined [35]. The authors described that the overall level of agreement of this software with subjective scales ranged from fair to moderate. Therefore, it was suggested to consider clinician experience and patient values additionally in clinical decision-making along with objective methods to achieve more satisfactory outcomes.

In the present study, the subjective estimates of the four linear dimensions on either side of the breast enabled a simplified breast shape and symmetry analysis [14]. The simplicity of this approach raises the prospect of its future widespread application. The comparison with the linear distances measured using the Vectra Camera System provided valid data on the accuracy and reproducibility of the method despite all possible errors that might occur.

It is expected that various methods, including the subjective method as well as 2D and 3D breast analyses, will continue to be used despite their individual advantages and disadvantages. In particular, while 3D analysis seems to be one of the most modern approaches and 2D tape measurements remain to have a low cost and be easily conducted, subjective methods are anticipated to continue to be applied in breast aesthetics despite all possible limitations owing to their ubiquitous availability and fast and simple application.

## Conclusion

The error assessment reveals that the reproducibility and accuracy of the subjective method vary significantly and inconsistently between examiners in comparison to the objective data obtained with an automated system. 

## Notes

### Acknowledgements

We thank Dr Wolfgang Reimers for providing support in the statistical analysis of the data and Dr Holger Hofheinz for providing access to the 3D Vectra Imaging System at the Klinik am Rhein, Düsseldorf.

### Ethical approval

All procedures performed in the study were conducted in accordance with the ethical standards of the institutional and national research committees and with the 1964 Helsinki Declaration and its later amendments or comparable ethical standards.

### Competing interests

The authors declare that they have no competing interests. This study is an independent investigation into subjective and objective assessments of linear breast dimensions.

## Figures and Tables

**Table 1:**
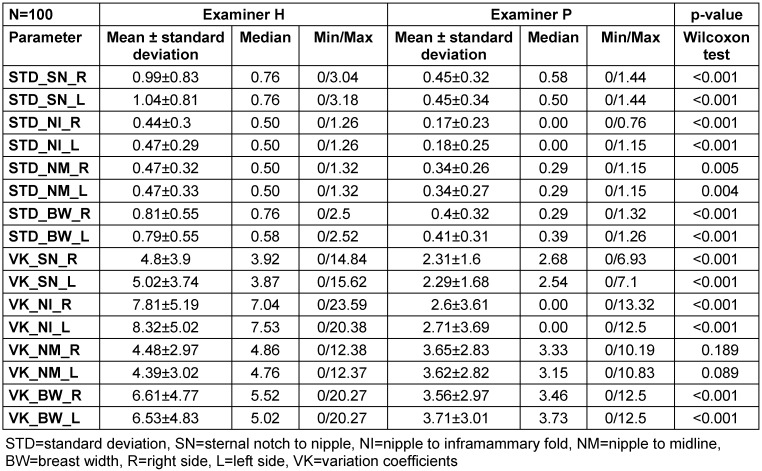
Intra-individual standard deviations, variation coefficients and p-values

**Table 2:**
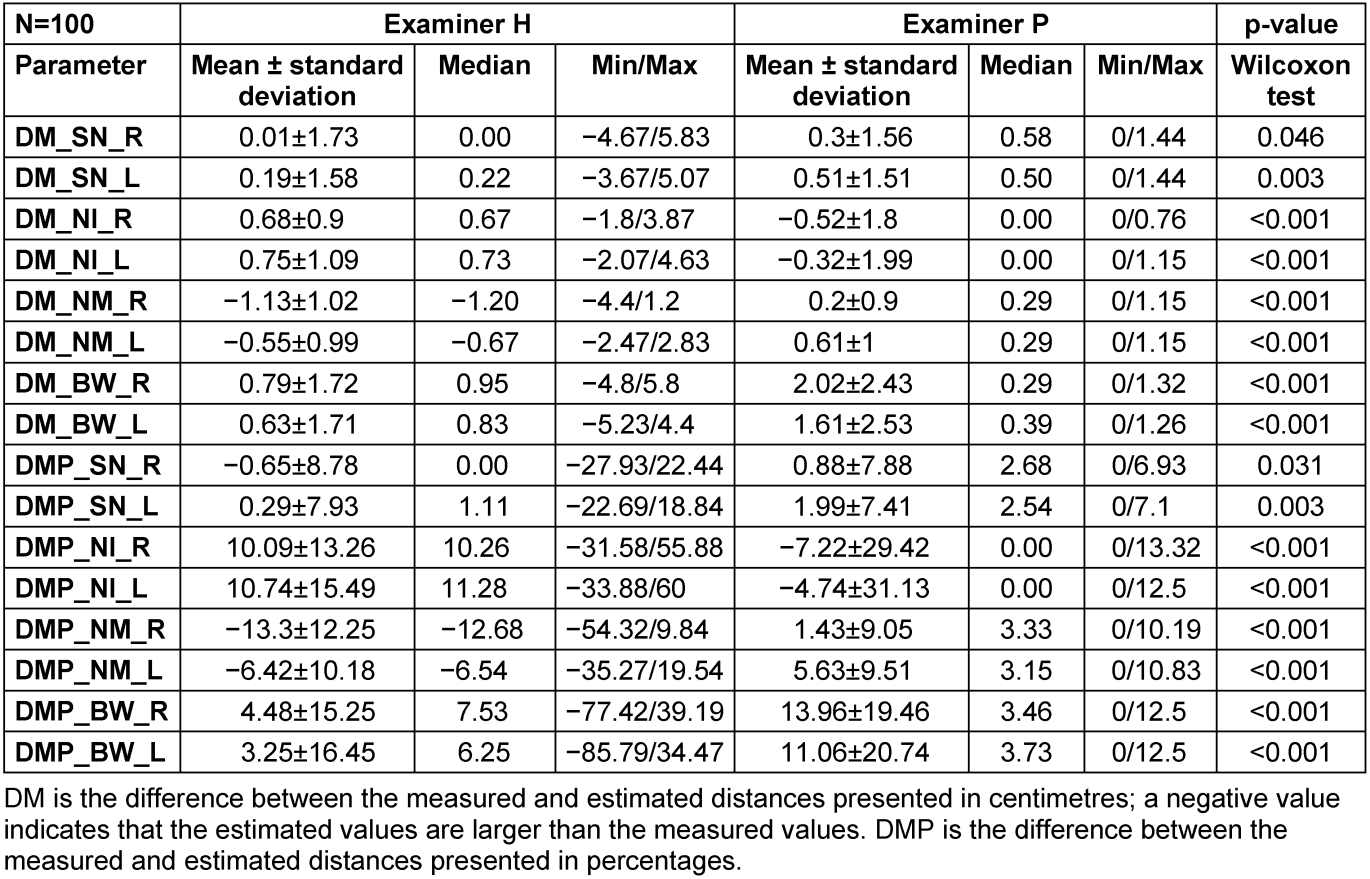
Differences between the estimated and measured distances and between the examiners

**Table 3:**
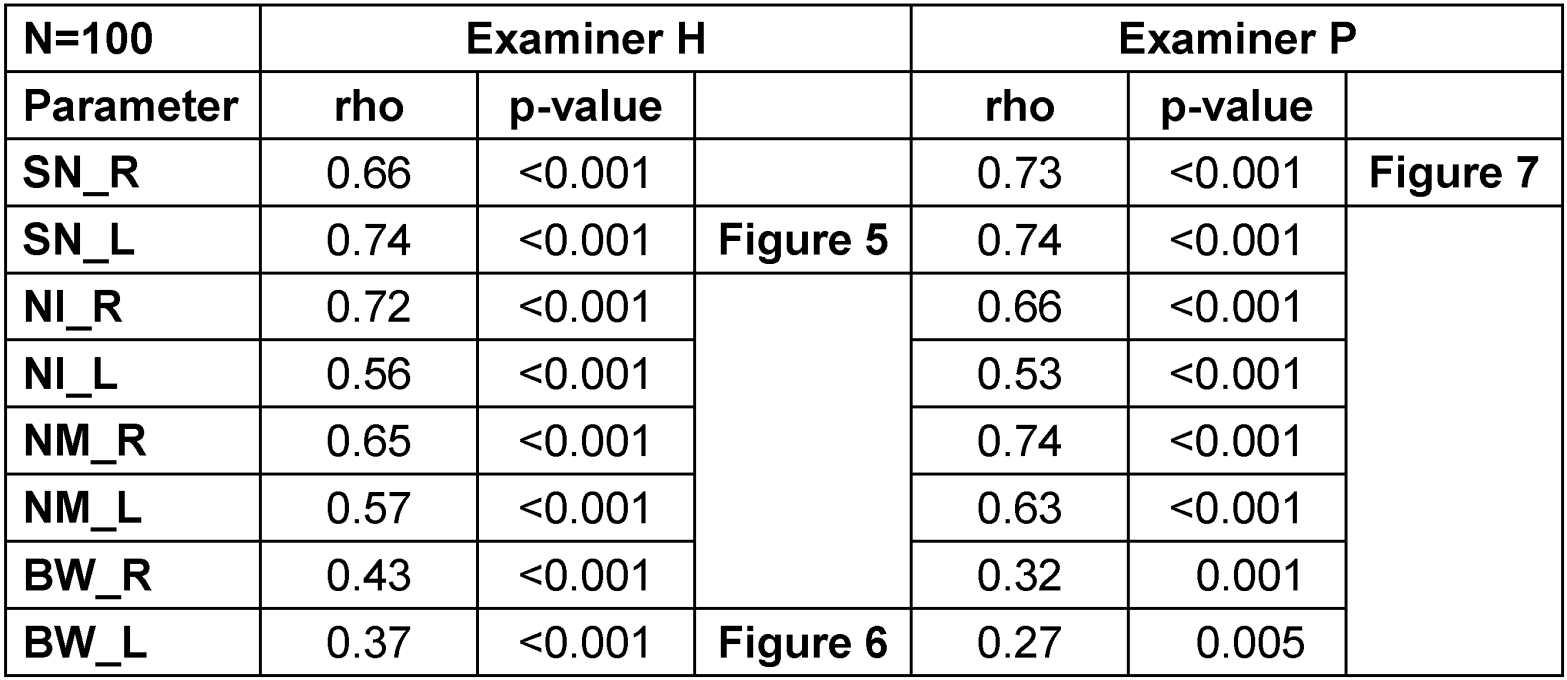
Correlation coefficients between the measured and estimated distances for both examiners
